# Lead compounds for the development of SARS-CoV-2 3CL protease inhibitors

**DOI:** 10.1038/s41467-021-22362-2

**Published:** 2021-04-01

**Authors:** Sho Iketani, Farhad Forouhar, Hengrui Liu, Seo Jung Hong, Fang-Yu Lin, Manoj S. Nair, Arie Zask, Yaoxing Huang, Li Xing, Brent R. Stockwell, Alejandro Chavez, David D. Ho

**Affiliations:** 1 Aaron Diamond AIDS Research Center, Columbia University Irving Medical Center, New York, NY USA; 2 Department of Microbiology and Immunology, Columbia University Irving Medical Center, New York, NY USA; 3 Herbert Irving Comprehensive Cancer Center, Columbia University Irving Medical Center, New York, NY USA; 4 Department of Chemistry, Columbia University, New York, NY USA; 5 Department of Pathology and Cell Biology, Columbia University Irving Medical Center, New York, NY USA; 6 WuXi AppTec, Cambridge, MA USA; 7 Department of Biological Sciences, Columbia University, New York, NY USA

**Keywords:** X-ray crystallography, Virology, SARS-CoV-2, Molecular medicine

## Abstract

We report the identification of three structurally diverse compounds – compound 4, GC376, and MAC-5576 – as inhibitors of the SARS-CoV-2 3CL protease. Structures of each of these compounds in complex with the protease revealed strategies for further development, as well as general principles for designing SARS-CoV-2 3CL protease inhibitors. These compounds may therefore serve as leads for the basis of building effective SARS-CoV-2 3CL protease inhibitors.

## Abstract (web summary)

The essential SARS-CoV-2 3CL protease is of interest as a drug target. Here, the authors identify three 3CL inhibitors and characterize them both in vitro and with a cell-based assay, and they also present the inhibitor-bound 3CL crystal structures, which may allow for the design of improved compounds.

## Introduction

As the etiologic agent of COVID-19, SARS-CoV-2 has resulted in millions of deaths and caused rampant economic damage worldwide^1,2^. While some treatments have been identified, their clinical efficacy is low or require delivery within a narrow treatment window, making continued research for additional therapeutics essential^3,4^. Similar to other coronaviruses, SARS-CoV-2 encodes an essential 3CL protease (3CL^pro^ or M^pro^) that processes its polyproteins, which has garnered interest as a target for potential viral inhibitors^5,6^. Here, we describe a series of compounds with inhibitory activity against SARS-CoV-2 3CL^pro^ and determine their structures in complex with the protease. These data provide general insights into the design of 3CL protease inhibitors, along with potential avenues by which these classes of compounds can be further developed.

## Results

### Identification of SARS-CoV-2 3CL protease inhibitors

**Fig. 1:**
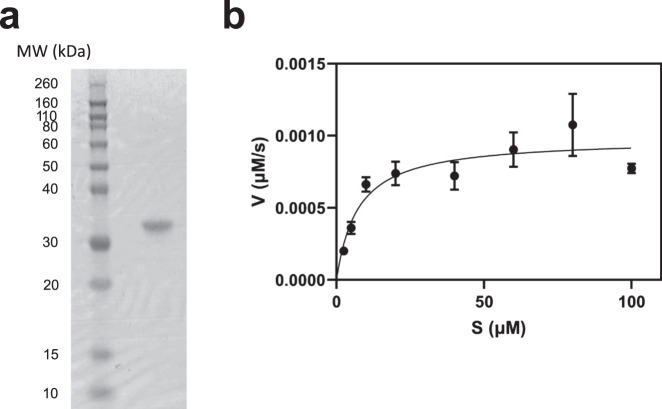
Production of native SARS-CoV-2 3CL protease in *E. coli*. **a** The purified protease was ran on SDS-PAGE to confirm size and purity. **b** Confirmation of enzymatic activity of SARS-CoV-2 3CL protease by quantification of cleavage of a fluorogenic peptide substrate. *K*_m_ was 7.22 ± 2.48 µM, *V*_max_ was 1 ± 0.08 nM/s, and catalytic efficiency (*k*_cat_/*K*_m_) was 6,925 M^−1^s^−1^. Data are shown as mean ± s.e.m. for three independent biological replicates. MW molecular weight.

**Fig. 2:**
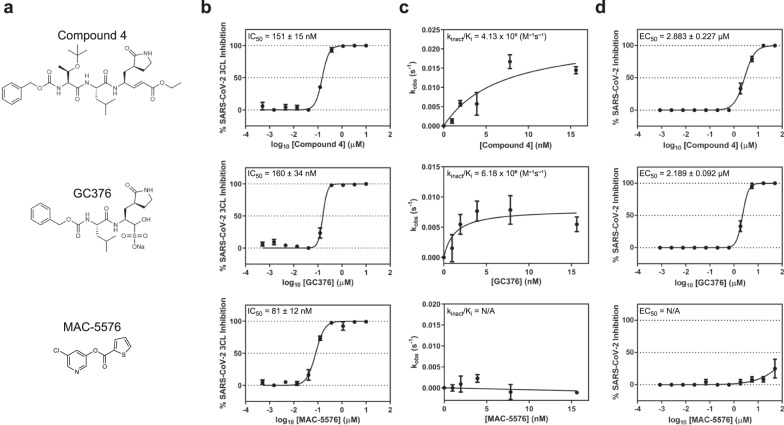
Inhibition of SARS-CoV-2 3CL protease by compound 4, GC376, and MAC-5576. **a** Chemical structures of the three compounds in this study. **b** Inhibition of purified native SARS-CoV-2 3CL protease by each compound. **c** Kinetics of inhibition of SARS-CoV-2 3CL protease by each compound. **d** Inhibition of SARS-CoV-2 viral replication by each compound. Data are shown as mean ± s.e.m. for two or three technical replicates for **b**, two technical replicates for **c**, and for three technical replicates for **d**. IC_50_ and EC_50_ values denote mean ± s.e.m. for two independent biological replicates.

We hypothesized that previously identified SARS-CoV 3CL protease inhibitors may also be effective against the SARS-CoV-2 3CL, given the conservation between the two proteases (96% amino acid identity)^1,2^. To study such compounds, we first purified the native SARS-CoV-2 3CL protease from *Escherichia* *coli* and confirmed that it had functional enzymatic activity in an in vitro biochemical assay (Fig. 1a, b). Using this assay to report SARS-CoV-2 3CL protease activity, we identified three diverse compounds of interest: compound 4^7^, GC376^8^, and MAC-5576^9^ (Fig. 2a). These compounds demonstrated inhibition of the protease with IC_50_ values (mean ± s.e.m.) of 151 ± 15, 160 ± 34, and 81 ± 12 nM, respectively (Fig. 2b). We further characterized these compounds by conducting enzyme kinetic studies to determine the inactivation rate (*k*_inact_/*K*_i_) for each compound (Fig. 2c). Compound 4 had a *k*_inact_/*K*_i_ of 4.13 × 10^5^ M^−1^s^−1^ and GC376 had a *k*_inact_/*K*_i_ of 6.18 × 10^6^ M^−1^s^−1^, but we did not observe time-dependent inhibition by MAC-5576.

We then tested these compounds for inhibition of SARS-CoV-2 viral replication. We found that compound 4 and GC376 could block viral infection in Vero-E6 cells in a cytopathic effect reduction assay (EC_50_ values (mean ± s.d.): 2.88 ± 0.23 and 2.19 ± 0.01 µM, respectively), whereas MAC-5576 did not (Fig. 2d). We confirmed that these compounds did not result in cytotoxicity to the cells at the tested concentrations (Supplementary Fig. 1).

### Crystal structures of 3CL^pro^ with protease inhibitors

As the three compounds exhibited inhibitory activity against the SARS-CoV-2 3CL^pro^, we proceeded to solve the crystal structure of the ligand-free 3CL protease alone and of each of these compounds in complex with the protease to understand their mechanism of binding as well as to guide future structure-based optimization efforts. We note that while MAC-5576 did not exhibit activity in the cellular assay, its low molecular weight and reasonable biochemical activity prompted us to pursue its crystallization as well, as our goal was to broadly investigate inhibitory scaffolds for the SARS-CoV-2 3CL protease. Crystals were obtained (see Methods for detailed information) and structures at 1.85, 1.94, 1.83, 1.73 Å resolution limits for ligand-free 3CL^pro^ and 3CL^pro^ bound to compound 4, GC376, and MAC-5576, respectively, were solved (Fig. 2, see Table 1 for statistics).

**Table 1:** Data collection and refinement statistics of ligand-free SARS-CoV-2 3CL protease and 3CL protease bound to compound 4, GC376, and MAC-5576.

	Ligand-free 3CL (PDB: 7JST)	3CL with compound 4 (PDB: 7JT7)	3CL with compound 4 (PDB: 7JW8)	3CL with GC376 (PDB: 7JSU)	3CL with MAC-5576 (PDB: 7JT0)
Data collection					
Space group	*C*2	*C*2	*P*1	*C*2	*C*2
Cell dimensions					
*a*, *b*, *c* (Å)	98.7, 82.0, 51.8	97.2, 81.9, 54.2	63.5, 67.8, 93.6	98.8, 80.2, 52.0	98.3, 82.5, 51.8
α, β, γ (°)	90, 114.9, 90	90, 117.1, 90	75.2, 79.3, 67.9	90, 114.3, 90	90, 114.83, 90
Resolution (Å)	60.4–1.85 (1.88–1.85)^a^	59.5–1.94 (1.98–1.94)^a^	90.03–1.84 (1.86–1.84)^a^	59.9–1.83 (1.86–1.83)^a^	60.58–1.73 (1.76–1.73)^a^
*R*_merge_ (%)	7.2 (65.7)	18.2 (58.4)	13.2 (76.5)	14.7 (60.5)	4.1 (68.8)
I/σI	13.4 (2.1)	10.2 (3.0)	5.7 (1.8)	12.7 (2.2)	23.5 (2.3)
Completeness (%)	98.9 (97.4)	98.8 (99.0)	95.3 (94.0)	98.5 (96.0)	99.2 (88.3)
Redundancy	6.8 (6.5)	6.7 (6.5)	3.6 (3.1)	6.9 (6.1)	6.8 (6.6)
CC1/2	0.99 (0.95)	0.99 (0.92)	0.99 (0.89)	0.99 (0.93)	1.00 (0.90)
Refinement					
Resolution (Å)	47.0–1.85 (1.88–1.85)^a^	48.20–1.94 (1.98–1.94)^a^	90.03–1.84 (1.86–1.84)^a^	47.38–1.83 (1.86–1.83)	47.03–1.73 (1.75–1.73)
No. reflections	31,709 (3219)	27,502 (2673)	113,161 (11,324)	31,971 (3134)	38,879 (3899)
*R*_work_/*R*_free_ (%)	16.8 (25.2)/19.6 (30.2)	17.4 (21.7)/22.5 (26.5)	18.3 (27.6)/22.7 (33.3)	17.4 (25.0)/20.6 (29.6)	16.6 (26.6)/19.2 (27.5)
Ramachantran plot (%)					
Outliers	0.00	0.00	0.16	0.00	0.00
Allowed	1.00	1.64	1.81	1.30	2.00
Favored	99.00	98.36	98.02	98.70	98.00
No. atoms					
Protein	2329	2347	9452	2340	2317
Ligand/ion	5	45	208	39	12
Water	128	274	1048	183	241
B-factors					
Protein	50.0	32.5	29.6	40.5	41.6
Ligand/ion	52.6	32.1	33.8	40.0	41.1
Water	53.5	41.5	40.0	47.9	49.9
R.m.s deviations					
Bond lengths (Å)	0.006	0.006	0.006	0.006	0.006
Bond angles (°)	0.8	0.8	0.8	0.8	0.8

^a^Highest resolution shell is shown in parenthesis.

**Fig. 3:**
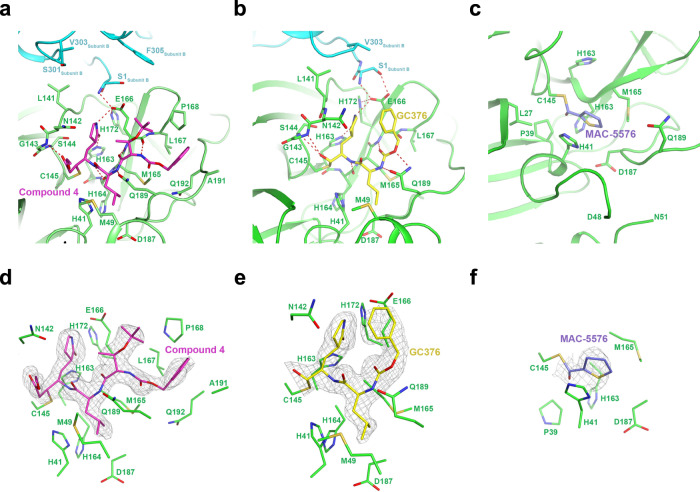
Crystal structures of inhibitors in complex with the SARS-CoV-2 3CL protease. Structure of compound 4 (**a**), GC376 (**b**), or MAC-5576 (**c**) bound to SARS-CoV-2 3CL^pro^. Protomer A is denoted in green and protomer B is denoted in cyan. The Fo–Fc omit map, contoured at 3σ (gray), for compound 4 (**d**), GC376 (**e**), and MAC-5576 (**f**).

The X-ray crystal structures revealed that all three compounds bind covalently to the catalytically active Cys145 residue within the substrate-binding pocket of the protease. We observed distinct mechanisms by which these compounds acted on this residue. Compound 4 functioned in a similar binding mode as other reported compounds, covalently modifying Cys145 through Michael addition (Fig. 3a)^5^. For GC376, the bisulfite adduct was converted to an aldehyde as previously reported, allowing it to then react with Cys145 through nucleophilic addition and hemithioacetal formation (Fig. 3b)^8^. MAC-5576 also covalently modified Cys145 by nucleophilic linkage, which was somewhat unexpected, given that we did not observe time-dependent inhibition by this compound (Fig. 3c). We observed weaker density in the S4 site for compound 4 (Fig. 3d) and in the S3 site for GC376 (Fig. 3e) as compared to other regions of each inhibitor. For MAC-5576, we found that the overall electron density is weak, and that the optimal modeling was achieved when the occupancy was set to 0.5, supporting that it may bind reversibly (Fig. 3f).

### Structural insights into the design of 3CL protease inhibitors

**Fig. 4:**
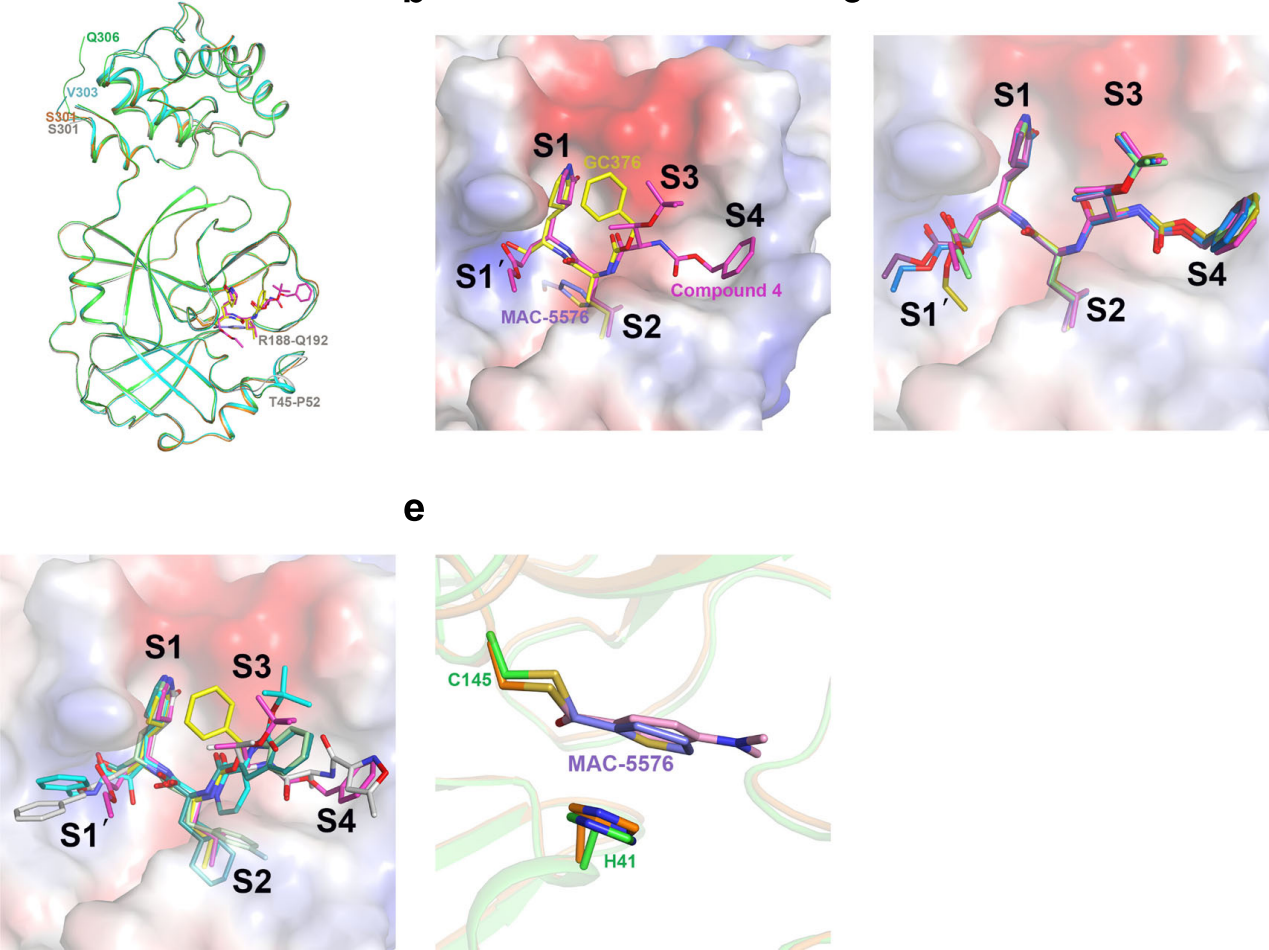
Overlays of the crystal structures. **a** Crystal structure of the ligand-free 3CL^pro^ (gray), in complex with, compound 4 (green for 3CL^pro^ and magenta for compound 4), GC376 (cyan for 3CL^pro^ and yellow for GC376), and MAC-5576 (orange for 3CL^pro^ and purple for MAC-5576). One protomer for each structure is shown, with the inhibitors shown with stick models. The terminal residue of each structure, as well as two stretches of residues near the binding site that exhibit local conformational change between the ligand-free and inhibitor-bound structures are labeled. **b** Overlay of all three compounds in the substrate-binding pocket of the 3CL protease. **c** Overlay of five molecules of compound 4, one from space group *C*2 (magenta) and four from four promoters in space *P*1 (light green, purple, marine, dark gold) for compound 4 bound to promoter A, B, C, and D of space group *P*1, respectively. **d** Comparison of the binding modes of compound 4 and GC376 with other peptide-like inhibitors. Compound 4 (magenta) and GC376 (yellow) were overlaid with previously identified compounds, compound 13b (cyan, PDB: 6Y2F), compound 11a (dark green, PDB: 6LZE), compound 11b (light green, PDB: 6M0K), and N3 (white; PDB: 7BQY). **e** Comparison of the binding modes of MAC-5576 with XP-59. MAC-5576 (purple) bound to the SARS-CoV-2 3CL protease (orange) was overlaid with XP-59 (pink) bound to the SARS-CoV 3CL protease (green, PDB: 2V6N).

As we solved the structures for multiple compounds, we hypothesized that general principles for the design of SARS-CoV-2 3CL protease inhibitors could be identified. We first overlaid all four crystal structures of the 3CL^pro^ with or without inhibitors (Fig. 4a). We observed local conformational changes, with Thr45 to Pro52 distinct from the ligand-free 3CL^pro^ in all three inhibitor-bound structures, whereas Arg188 to Gln192 differed only in the compound 4 and GC376-bound, but not MAC-5576-bound structures. We then overlaid each of the inhibitors in the substrate-binding pocket of the 3CL protease to find commonalities in their interactions (Fig. 4b). Most notably, we found that all of these compounds occupied the S2 site, with compound 4 and GC376 further anchored in the S1 site. The backbone NH of Gly143 points toward the ligand-binding pocket, forming hydrogen bonds with the carbonyl oxygen of the ethyl ester of compound 4, and the hemithioacetal of GC376 after the Cys145 addition to the original aldehyde, even though the former hydrogen bond is stronger than the latter. In both structures, the γ-lactam groups occupy the S1 site, and are strongly anchored by two hydrogen bonds with the side chains of His163 and Glu166. The isobutyl groups are favorably embedded in the hydrophobic S2 site, surrounded by the alkyl portion of the side chains of His41, Met49, His164, Met165, Asp187, and Gln189. Extending into the S3 pocket, the amide bonds of compound 4 and GC376 are stabilized by hydrogen bond interactions with the side chain of Gln189.

To further study the interaction of compound 4 with SARS-CoV-2 3CL^pro^, we determined and refined an additional crystal structure of the 3CL protease in complex with compound 4 in space group *P*1 at 1.84 Å resolution limits, in which there are four protomers in the asymmetric unit of the crystal, which is equivalent with the unit cell in this space group. Overlaying these protomers revealed that in particular, compound 4 exhibited significant flexibility in the S1′ region (Fig. 4c).

As several SARS-CoV-2 3CL protease inhibitors have been reported, we overlaid compound 4 and GC376 with these related substrate mimetic inhibitors (Fig. 4d)^5,6,10^. We found similar interactions between these compounds, suggesting that overall, the binding modes of this class share remarkable similarities. Specifically, they all have a γ-lactam occupying the S1 pocket, preserving the dual hydrogen bonds with His163 and Glu166. Furthermore, they commonly contain a hydrophobic moiety occupying the S2 site. The segment of the inhibitors from S1 to S2 align closely on top of each other. Variations of binding start to emerge in the S3 and S4 region, which exhibits high degrees of freedom in terms of structural diversity as well as conformational flexibility.

On the other hand, the binding of MAC-5576, as a non-peptidic small molecule, displays unique features that differ from that of compound 4 or GC376. We observed that the thiophene group forms π–π stacking with the His41 side chain imidazole, which undergoes a conformational rotation around its beta-carbon to align parallel to the thiophene, as compared to the other peptide-bound structures. Additionally, the side chain of Gln189 also shows notable conformational variation compared to those in the compound 4 and GC376 crystal structures, possibly in response to the specific hydrogen bond interactions induced by the respective ligands. Notably, the rotation of His41 has been reported previously in the crystal structure of a benzotriazole ester inhibitor (XP-59) in complex with the SARS-CoV 3CL protease (PDB:2V6N)^11^. An overlaid model of the crystal structures of MAC-5576 bound to SARS-CoV-2 3CL^pro^ and XP-59 bound to SARS-CoV 3CL^pro^ shows that both compounds have similar binding modes when covalently bound to Cys145, in which the thiophene of MAC-5576 and the phenyl ring of XP-59 almost overlap with each other, both engaging the His41 side chain via π-π stacking interactions (Fig. 4e).

## Discussion

In this study, we have identified compound 4, GC376, and MAC-5576 as inhibitors of the SARS-CoV-2 3CL protease. Each of these compounds displayed biochemical inhibition of the protease, and compound 4 and GC376 also inhibited the virus in a cell-based assay, whereas MAC-5576 did not (Fig. 2). We solved the crystal structures of these compounds complexed to the protease, confirming that each are covalent inhibitors (Fig. 3). Compound 4 and GC376 demonstrated similar interactions as other substrate mimetic inhibitors^5,6,10^, and MAC-5576 was similar to a previously identified small molecule inhibitor of SARS-CoV 3CL^pro 11^ (Fig. 4).

GC376 has been recently reported to be an inhibitor of the SARS-CoV-2 3CL^pro^, and the complex was solved by Ma et al. (PDB accession code 6WTT)^12^. Our results corroborate their findings, and we observe similar interactions in our solved structure. However, one notable difference lies in the S3 site, in which the benzyl group in our crystal structure points upward towards the solvent, while making a hydrophobic interaction with the lactam group. In contrast, the benzyl group of GC376, bound to each of the three 3CL protomers in the asymmetric unit (ASU) of their structure, is anchored in the hydrophobic pocket predominantly formed by Met165, Leu167, and Gln192. This observation, along with the observed weaker electron density in this region (Fig. 3e), suggests that this subsite could be modified for an improved inhibitor.

In solving the complex of compound 4 with 3CL protease in both space groups *C*2 and *P*1, we observed that the S1′ site demonstrated conformational flexibility (Fig. 4c). In addition, the S4 site demonstrated weaker electron density (Fig. 3d), suggesting that modifying the interaction of compound 4 with these two subsites could improve the compound’s inhibitory activity.

The finding that MAC-5576 was covalently linked to Cys145 in the crystal structure (Fig. 3c) but did not display time-dependent inhibition (Fig. 2c) suggests that it may be a reversible covalent inhibitor. The overall weaker electron density and optimal modeling with occupancy set to 0.5 for this structure supports the possibility of its reversible nature. However, it is possible that the lack of time-dependent inhibition, yet the observation of a clear covalent linkage in the crystal structure, is due to the differences in the conditions used for the two experiments. Further investigations into the mechanism of action of MAC-5576 may reveal a method for alleviating its lack of activity in inhibiting the virus (Fig. 2d).

The collective observations from the three inhibitors suggest that development of 3CL protease inhibitors may benefit from first establishing robust interactions within the S1, S2, and/or S1′ sites, before extending into the S3 and S4 sites. For these, and other compounds targeting the 3CL protease, there are ample opportunities to improve the inhibitory potencies against the 3CL^pro^ by designing compounds that exploit the accessible contact points to strengthen the ligand-protein interactions (Fig. 4d).

In summation, we have identified compound 4, GC376, and MAC-5576 as inhibitors of the SARS-CoV-2 3CL protease. Crystal structures of the compounds complexed to the protease suggested their mechanisms of action, as well as portended guidelines for the development of SARS-CoV-2 3CL protease inhibitors, which may aid in the future development of novel inhibitors to combat this virus.

## Methods

### Compounds

Compound 4 was synthesized using the synthesis route previously described, with the exception of using a sodium borohydride-cobaltous chloride reduction of the nitrile in the construction of the lactam, thus avoiding the high pressure hydrogenation in the original route^7,13^. GC376 was purchased from Aobious (Gloucester, MA, USA) and MAC-5576 was purchased from Maybridge (Cheshire, United Kingdom).

### Expression and purification of SARS-CoV-2 3CL protease

The SARS-CoV-2 3CL protease gene was codon optimized for bacterial expression and synthesized (Supplementary Table 1) (Twist Bioscience, San Francisco, CA, USA), then cloned into a bacterial expression vector (pGEX-5X-3, GE, Boston, MA, USA, gift from Yosef Sabo, Columbia University Irving Medical Center) which expresses the protease as a fusion construct with a N-terminal GST tag, followed by a Factor Xa cleavage site (pGEX-5X-3-SARS-CoV-2-3CL, deposited to Addgene as plasmid #168457). After confirmation by Sanger sequencing using the primers listed in Supplementary Table 2, the construct was transformed into BL21 (DE3) cells. These *E. coli* were inoculated and grown overnight as starter cultures, then used to inoculate larger cultures at a 1:100 dilution, which were then grown at 37 °C, 220 RPM until the OD reached 0.6–0.7. Expression of the protease was induced with the addition of 0.5 mM IPTG, and then the cultures were incubated at 16 °C, 180 RPM for 10 h. Cells were pelleted at 3580 × *g* for 15 min at 4 °C, resuspended in lysis buffer (20 mM Tris-HCl, pH 8.0, 300 mM NaCl), homogenized by sonication, then clarified by centrifuging at 25,000 × *g* for 1 h at 4 °C. The supernatant was mixed with Glutathione Sepharose resin (Sigma, St. Louis, MO, USA) and placed on a rotator for 2 h at 4 °C. The resin was then repeatedly washed by centrifugation at 3210 × *g* for 15 min at 4 °C, discarding of the supernatant, and then resuspension of the resin in fresh lysis buffer. After ten washes, the resin was resuspended in lysis buffer, and Factor Xa was added and incubated for 36 h at 4 °C on a rotator. The resin was centrifuged at 3210 × *g* for 15 min at 4 °C, and then the supernatant was collected and concentrated using a 10 kDa concentrator before being loaded onto a Superdex 10/300 GL column in 50 mM Tris-HCl, pH 7.5, 1 mM EDTA for further purification by size exclusion chromatography. The appropriate fractions were collected and pooled with a 10 kDa concentrator, and then the final product was assessed for quality by SDS-PAGE and measurement of biochemical activity.

### Measurement of SARS-CoV-2 3CL protease biochemical activity

The in vitro biochemical activity of the SARS-CoV-2 3CL protease was measured as previously described^5^. The fluorogenic peptide MCA-AVLQSGFR-Lys(DNP)-Lys-NH2, corresponding to the nsp4/nsp5 cleavage site in the virus, was synthesized (GL Biochem, Shanghai, China), then resuspended in DMSO to use as the substrate. Different concentrations of this substrate, ranging from 5 to 100 µM, were prepared in the assay buffer (50 mM Tris-HCl, pH 7.5, 1 mM EDTA) in a 96 well-plate. The protease was then added to each well at a concentration of 0.2 µM, and then fluorescence was continuously measured on a plate reader for 3 min. The catalytic efficiency of the protease was then calculated by nonlinear regression (GraphPad Prism, GraphPad Software, San Diego, CA, USA). For calculations, a 100% active enzyme was assumed.

### Measurement of SARS-CoV-2 3CL protease inhibition

Inhibition of the biochemical activity of the SARS-CoV-2 3CL protease was quantified as previously described with modifications^5^. Serial dilutions of the test compound were prepared in the assay buffer, and then incubated with 0.2 µM of the protease for 10 min at 37 °C. The substrate was then added at 20 µM per well, and then fluorescence was continuously measured on a plate reader for 3 min. Inhibition was then calculated by comparison to control wells with no inhibitor added. IC_50_ values were determined by nonlinear regression (GraphPad Prism). For calculations, a 100% active enzyme was assumed.

Kinetic parameters were determined as previously described^14^. Compounds were pre-incubated with the protease at differing timepoints at various concentrations to derive *k*_obs_, which were then used for the calculation of *k*_inact_ and *K*_i_ by nonlinear regression (GraphPad Prism).

### Measurement of SARS-CoV-2 viral inhibition

Stocks of SARS-CoV-2 strain 2019-nCoV/USA_WA1/2020 were propagated and titered in Vero-E6 cells. One day prior to the experiment, Vero-E6 cells were seeded at 30,000 cells/well in 96 well-plates. Serial dilutions of the test compound were prepared in cell media (EMEM + 10% FCS + penicillin/streptomycin), overlaid onto cells, and then virus was added to each well at an MOI of 0.2. Cells were incubated at 37 °C under 5% CO_2_ for 72 h and then viral cytopathic effect was scored in a blinded manner. Inhibition was calculated by comparison to control wells with no inhibitor added. EC_50_ values were determined by nonlinear regression (GraphPad Prism). Cells were confirmed as mycoplasma negative prior to use. All experiments were conducted in a biosafety level 3 (BSL-3) lab.

### Measurement of cellular cytotoxicity

Vero-E6 cells were incubated with the compound of interest for 48 h at 37 °C under 5% CO_2_ and then cellular cytotoxicity was determined with the XTT Cell Proliferation Assay Kit (ATCC) according to the manufacturer’s instructions.

### Crystallization, data collection, and structure determination

To generate the complex of SARS-CoV-2 3CL protease bound to compound 4, 50 µM of the 3CL protease was incubated with 500 µM of compound 4 in a buffer comprised of 50 mM Tris-HCl (pH 7.5), 1 mM EDTA, and 5% (v/v) glycerol for 1 h at 4 °C. This complex was then concentrated to 8.5 mg/mL using a 10 kDa concentrator, and initially subjected to extensive robotic screening at the High-Throughput Crystallization Screening Center of the Hauptman-Woodward Medical Research Institute (HWI) (https://hwi.buffalo.edu/high-throughput-crystallization-center/)^15^. The most promising crystal hits were then reproduced using the microbatch-under-oil method at 4 °C. Block-like crystals of 3CL^pro^ in complex with compound 4 appeared after a few days in the crystallization condition comprised of 0.1 M potassium nitrate, 0.1 M sodium acetate (pH 5), and 20% (w/v) PEG 1000 with protein to crystallization reagent at a 2:1 ratio. The crystals were subsequently transferred into the same crystallization reagent supplemented with 15% (v/v) glycerol and flash-frozen in liquid nitrogen. Plate-like crystals of 3CL^pro^ in complex with compound 4 were also produced using crystallization reagent comprising 0.1 M Bis-Tris (pH 6.5) and 20% (w/v) PEG MME 5000.

To obtain crystals of 3CL^pro^ in complex with GC376, crystals of ligand-free 3CL^pro^ were initially grown by using seeding method in a crystallization reagent comprised of 0.1 M sodium phosphate-monobasic, 0.1 M MES (pH 6), and 20% (w/v) PEG 4000. These crystals were subsequently soaked with 15 mM GC376, followed by flash-freezing of the crystals in the same reagent supplemented with 15% ethylene glycol.

To generate the complex of SARS-CoV-2 3CL protease bound to MAC-5576, 50 µM of the 3CL protease was incubated with 500 µM of MAC-5576 in a buffer comprised of 50 mM Tris-HCl (pH 7.5), 1 mM EDTA, and 5% (v/v) glycerol for 1 h at 4 °C. The complex was concentrated to 10 mg/mL using a 10 kDa concentrator, and then crystallized in the same conditions as those used for crystallization of ligand-free 3CL^pro^.

A native dataset was collected on each crystal of 3CL^pro^, alone (ligand-free), and in complex with compound 4 and GC376 at the NE-CAT24-ID-C beam line of Advanced Photon Source (APS) at Argonne National Laboratory, and the NE-CAT 24-ID-E beam line of APS was used for data collection on crystals of 3CL-MAC-5576. Crystals of ligand-free 3CL^pro^ and in complex with compound 4 in space group *C*2 and *P*1, GC376, and MAC-5576 diffracted the X-ray beam to resolution 1.85, 1.94, 1.84, 1.83, 1.73 Å, respectively. The images were processed and scaled in space group *C*2 using XDS^16^. The structure of 3CL^pro^ with compound 4 in space group *C*2 was determined by molecular replacement (MR) method using program MOLREP^17^ and the crystal structure of 3CL^pro^ in complex with inhibitor N3 (PDB id: 6LU7)^5^ was used as a search model. The structure of 3CL^pro^ with compound 4 in space group *P*1 was also determined by MR method and the refined model of 3CL^pro^ with compound 4 in space group *C*2 was used as the search model. The geometry of each crystal structure was subsequently fixed and the corresponding inhibitor was modeled in by XtalView^18^ and Coot^19^, and refined using PHENIX^20^. The mapping of electrostatic potential surfaces was generated in PyMOL with the APBS plug-in^21^. There is one protomer of 3CL^pro^ complex in the asymmetric unit of each crystal of space group *C*2, and there are four protomers of 3CL^pro^ bound to compound 4 in each unit cell of space group *P*1. The crystallographic statistics are shown in Table 1.

### Reporting summary

Further information on experimental design is available in the Nature Research Reporting Summary linked to this paper.

## Supplementary information

Supplementary Information

Reporting summary

## Data Availability

The SARS-CoV-2 3CL^pro^ bacterial expression vector utilized in this study has been deposited to Addgene as plasmid #168457. Structural data for the ligand-free SARS-CoV-2 3CL protease and 3CL^pro^ in complex with compound 4 in space group *C*2 and *P*1, GC376, and MAC-5576 have been deposited in the Protein Data Bank (PDB) under accession codes 7JST, 7JT7, 7JW8, 7JSU, and 7JT0, respectively. Overlays in Fig. 4d, e were made using previously deposited structures in PDB, available under accession codes 6Y2F (SARS-CoV-2 3CL^pro^ with compound 13b), 6LZE (SARS-CoV-2 3CL^pro^ with compound 11a), 6M0K (SARS-CoV-2 3CL^pro^ with compound 11b), 7BQY (SARS-CoV-2 3CL^pro^ with N3), and 2V6N (SARS-CoV 3CL^pro^ with XP-59). Source data are provided with this paper.

## Source data

Source Data

## References

[1] Wu F (2020). A new coronavirus associated with human respiratory disease in China. Nature.

[2] Zhou P (2020). A pneumonia outbreak associated with a new coronavirus of probable bat origin. Nature.

[3] Beigel, J. H. et al. Remdesivir for the Treatment of Covid-19—Preliminary Report. *N. Engl. J. Med*. 10.1056/NEJMoa2007764 (2020). 10.1056/NEJMc2022236 32649078

[4] Wang Y (2020). Remdesivir in adults with severe COVID-19: a randomised, double-blind, placebo-controlled, multicentre trial. Lancet.

[5] Jin, Z. et al. Structure of M(pro) from SARS-CoV-2 and discovery of its inhibitors. *Nature*, 10.1038/s41586-020-2223-y (2020). 10.1038/s41586-020-2223-y 32272481

[6] Zhang L (2020). Crystal structure of SARS-CoV-2 main protease provides a basis for design of improved alpha-ketoamide inhibitors. Science.

[7] Yang S (2006). Synthesis, crystal structure, structure-activity relationships, and antiviral activity of a potent SARS coronavirus 3CL protease inhibitor. J. Med. Chem..

[8] Kim Y (2012). Broad-spectrum antivirals against 3C or 3C-like proteases of picornaviruses, noroviruses, and coronaviruses. J. Virol..

[9] Blanchard JE (2004). High-throughput screening identifies inhibitors of the SARS coronavirus main proteinase. Chem. Biol..

[10] Dai W (2020). Structure-based design of antiviral drug candidates targeting the SARS-CoV-2 main protease. Science.

[11] Verschueren KH (2008). A structural view of the inactivation of the SARS coronavirus main proteinase by benzotriazole esters. Chem. Biol..

[12] Ma C (2020). Boceprevir, GC-376, and calpain inhibitors II, XII inhibit SARS-CoV-2 viral replication by targeting the viral main protease. Cell Res..

[13] Zhai Y (2015). Cyanohydrin as an anchoring group for potent and selective inhibitors of enterovirus 71 3C protease. J. Med. Chem..

[14] Obach RS, Walsky RL, Venkatakrishnan K (2007). Mechanism-based inactivation of human cytochrome p450 enzymes and the prediction of drug-drug interactions. Drug Metab. Dispos..

[15] Luft JR (2003). A deliberate approach to screening for initial crystallization conditions of biological macromolecules. J. Struct. Biol..

[16] Kabsch W (2010). Integration, scaling, space-group assignment and post-refinement. Acta. Crystallogr. D. Biol. Crystallogr..

[17] Vagin A, Teplyakov A (2010). Molecular replacement with MOLREP. Acta. Crystallogr. D. Biol. Crystallogr..

[18] McRee DE (1999). XtalView/Xfit–A versatile program for manipulating atomic coordinates and electron density. J. Struct. Biol..

[19] Emsley P, Lohkamp B, Scott WG, Cowtan K (2010). Features and development of Coot. Acta. Crystallogr. D. Biol. Crystallogr..

[20] Adams PD (2010). PHENIX: a comprehensive Python-based system for macromolecular structure solution. Acta. Crystallogr. D. Biol. Crystallogr..

[21] Baker NA, Sept D, Joseph S, Holst MJ, McCammon JA (2001). Electrostatics of nanosystems: application to microtubules and the ribosome. Proc. Natl Acad. Sci. USA.

