# Author Correction: Lead compounds for the development of SARS-CoV-2 3CL protease inhibitors

**DOI:** 10.1038/s41467-021-23082-3

**Published:** 2021-05-05

**Authors:** Sho Iketani, Farhad Forouhar, Hengrui Liu, Seo Jung Hong, Fang-Yu Lin, Manoj S. Nair, Arie Zask, Yaoxing Huang, Li Xing, Brent R. Stockwell, Alejandro Chavez, David D. Ho

**Affiliations:** 1 Aaron Diamond AIDS Research Center, Columbia University Irving Medical Center, New York, NY USA; 2 Department of Microbiology and Immunology, Columbia University Irving Medical Center, New York, NY USA; 3 Herbert Irving Comprehensive Cancer Center, Columbia University Irving Medical Center, New York, NY USA; 4 Department of Chemistry, Columbia University, New York, NY USA; 5 Department of Pathology and Cell Biology, Columbia University Irving Medical Center, New York, NY USA; 6 WuXi AppTec, Cambridge, MA USA; 7 Department of Biological Sciences, Columbia University, New York, NY USA

**Keywords:** X-ray crystallography, Virology, SARS-CoV-2, Molecular medicine

Correction to: *Nature Communications* 10.1038/s41467-021-22362-2, published online 1 April 2021.

The original version of this Article omitted the following from the Acknowledgements: Part of the research was conducted at the Northeastern Collaborative Access Team beamlines, which are funded by the National Institute of General Medical Sciences from the National Institutes of Health (P30 GM124165). This has now been corrected in both the PDF and HTML versions of the Article.

