# Supplementary material for: Lead compounds for the development of SARS-CoV-2 3CL protease inhibitors
Source: Nat Commun. 2021 Apr 1;12:2016. doi: 10.1038/s41467-021-22362-2 (PMC8016852; doi:10.1038/s41467-021-22362-2)
Supplement: Supplementary file 1 — Supplementary Information [file 41467_2021_22362_MOESM1_ESM.pdf]

Supplementary Information for “Lead compounds for the development of SARS-CoV-2 3CL protease inhibitors”

Sho Iketani[1, 2], Farhad Forouhar[3], Hengrui Liu[4], Seo Jung Hong[5], Fang-Yu Lin[6], Manoj S. Nair[1], Arie Zask[7], Yaoxing Huang[1], Li Xing[6], Brent R. Stockwell[4, 7*], Alejandro Chavez[5*], David D. Ho[1*]

[1] Aaron Diamond AIDS Research Center, Columbia University Irving Medical Center, New York, NY 10032, USA

[2] Department of Microbiology and Immunology, Columbia University Irving Medical Center, New York, NY 10032, USA

[3] Herbert Irving Comprehensive Cancer Center, Columbia University Irving Medical Center, New York, NY 10032, USA

[4] Department of Chemistry, Columbia University, New York, NY 10027, USA

[5] Department of Pathology and Cell Biology, Columbia University Irving Medical Center, New York, NY 10032, USA

[6] WuXi AppTec, Cambridge, MA 02142, USA

[7] Department of Biological Sciences, Columbia University, New York, NY 10027, USA

* Corresponding authors

Supplementary Fig 1. Cytotoxicity of compounds in Vero-E6 cells. Vero-E6 cells were incubated with serial dilutions of each compound for 48 h and then tested for cytotoxicity. Data are shown as mean ± s.e.m. for three experimental replicates.

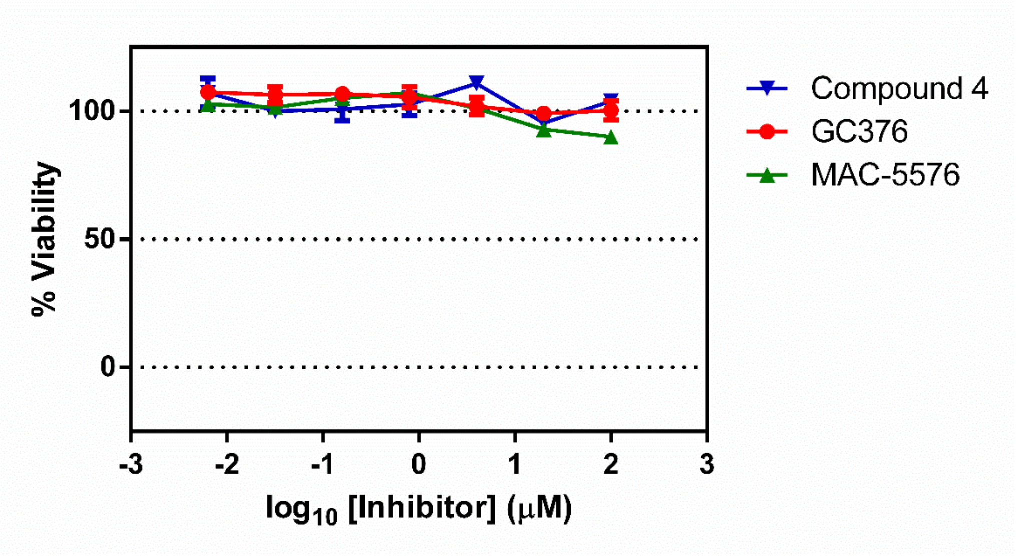

Supplementary Table 1. Bacterial codon optimized SARS-CoV-2 3CL protease sequence used in this study.

TCAGGGTTCCGCAAGATGGCGTTTCCGTCGGGAAAGGTGGAAGGCTGCATGGTCCAGGTGAC
CTGCGGCACTACGACGTTGAATGGCCTGTGGTTAGACGATGTCGTATATTGCCCGCGTCACGT
CATTTGTACGTCAGAGGATATGCTGAATCCCAACTACGAGGACTTGTTAATCCGCAAAAGTAAC
CACAACTTTCTGGTGCAAGCAGGGAACGTACAGCTGCGCGTGATCGGGCACTCGATGCAGAAC
TGCGTTCTCAAACTGAAAGTAGACACTGCAAACCCCAAAACGCCGAAATACAAATTCGTCCGTA
TCCAGCCGGGCCAAACCTTCTCCGTTTTGGCGTGCTATAACGGGAGCCCCTCGGGCGTGTATC
AGTGCGCGATGCGCCCGAACTTTACAATCAAAGGGTCGTTCTTGAACGGCAGTTGCGGAAGCG
TGGGCTTCAATATCGACTACGATTGCGTTAGTTTCTGCTATATGCATCACATGGAGCTCCCGAC
CGGCGTGCACGCCGGTACTGATCTTGAGGGGAATTTCTACGGGCCATTCGTAGATCGTCAGAC
CGCGCAGGCGGCAGGGACCGATACCACCATCACCGTGAACGTACTCGCGTGGCTGTATGCAG
CAGTGATCAACGGTGATCGCTGGTTCCTGAACCGCTTCACAACGACCCTCAACGATTTCAATTT
AGTCGCCATGAAATATAACTACGAGCCCTTGACGCAGGATCACGTGGATATCCTCGGCCCGCT
GAGTGCGCAGACGGGGATCGCGGTGCTGGACATGTGCGCGAGCCTGAAGGAGCTTTTACAGA
ACGGCATGAACGGTCGCACTATCTTAGGCAGCGCACTGCTGGAAGACGAGTTCACCCCGTTCG
ACGTAGTACGCCAGTGTAGCGGAGTAACCTTTCAGTGA

Supplementary Table 2. Primers used for Sanger sequencing of the SARS-CoV-2 3CL protease bacterial expression vector.

Name	Sequence
SI-AC018	TTGACAATTAATCATCGGCT
PAC3830	ATATAGCATGGCCTTTGCAG
PAC4378	GAGCTGCATGTGTCAGAGGT
