# Supplementary material for: Lead compounds for the development of SARS-CoV-2 3CL protease inhibitors
Source: Nat Commun. 2021 Apr 1;12:2016. doi: 10.1038/s41467-021-22362-2 (PMC8016852; doi:10.1038/s41467-021-22362-2)
Supplement: Supplementary file 2 — Reporting summary [file 41467_2021_22362_MOESM2_ESM.pdf]

nature research

Corresponding author(s): David D. Ho, Alejandro Chavez, Brent R. Stockwell

Last updated by author(s): Mar 6, 2021

Reporting Summary

Nature Research wishes to improve the reproducibility of the work that we publish. This form provides structure for consistency and transparency in reporting. For further information on Nature Research policies, see our Editorial Policies and the Editorial Policy Checklist.

Statistics

For all statistical analyses, confirm that the following items are present in the figure legend, table legend, main text, or Methods section.

n/a	Confirmed	
☐	☒	The exact sample size (n) for each experimental group/condition, given as a discrete number and unit of measurement
☐	☒	A statement on whether measurements were taken from distinct samples or whether the same sample was measured repeatedly
☒	☐	The statistical test(s) used AND whether they are one- or two-sided<br>*Only common tests should be described solely by name; describe more complex techniques in the Methods section.*
☒	☐	A description of all covariates tested
☒	☐	A description of any assumptions or corrections, such as tests of normality and adjustment for multiple comparisons
☐	☒	A full description of the statistical parameters including central tendency (e.g. means) or other basic estimates (e.g. regression coefficient) AND variation (e.g. standard deviation) or associated estimates of uncertainty (e.g. confidence intervals)
☒	☐	For null hypothesis testing, the test statistic (e.g. F, t, r) with confidence intervals, effect sizes, degrees of freedom and P value noted<br>*Give P values as exact values whenever suitable.*
☒	☐	For Bayesian analysis, information on the choice of priors and Markov chain Monte Carlo settings
☒	☐	For hierarchical and complex designs, identification of the appropriate level for tests and full reporting of outcomes
☒	☐	Estimates of effect sizes (e.g. Cohen's d, Pearson's r), indicating how they were calculated

Our web collection on statistics for biologists contains articles on many of the points above.

Software and code

Policy information about availability of computer code

Data collection	None
Data analysis	GraphPad Prism (Version 9.0.0), XtalView (Version 4.1), PHENIX (Version 1.17.1), MOLREP (from CCP4-7.0), Coot (Version 0.8.9.2), PyMOL (Version 2.3.4), APBS (Version 3.0.0)

For manuscripts utilizing custom algorithms or software that are central to the research but not yet described in published literature, software must be made available to editors and reviewers. We strongly encourage code deposition in a community repository (e.g. GitHub). See the Nature Research guidelines for submitting code & software for further information.

Data

Policy information about availability of data

All manuscripts must include a data availability statement. This statement should provide the following information, where applicable:

- Accession codes, unique identifiers, or web links for publicly available datasets
- A list of figures that have associated raw data
- A description of any restrictions on data availability

Structural data for the ligand-free SARS-CoV-2 3CL protease and 3CLpro in complex with compound 4 in space group C2 and P1, GC376, and MAC-5576 have been deposited in the Protein Data Bank (PDB) under accession codes 7JST (https://doi.org/10.2210/pdb7JST/pdb), 7JT7 (https://doi.org/10.2210/pdb7JT7/pdb), 7JW8 (https://doi.org/10.2210/pdb7JW8/pdb), 7JSU (https://doi.org/10.2210/pdb7JSU/pdb), and 7JT0 (https://doi.org/10.2210/pdb7JT0/pdb), respectively. Overlays in Fig. 4d and 4e were made using previously deposited structures in PDB, available under accession codes 6Y2F (SARS-CoV-2 3CLpro with compound 13b, http://doi.org/10.2210/pdb6Y2F/pdb), 6LZE (SARS-CoV-2 3CLpro with compound 11a, http://doi.org/10.2210/pdb6LZE/pdb), 6M0K (SARS-CoV-2 3CLpro with compound 11b, http://doi.org/10.2210/pdb6M0K/pdb), 7BQY (SARS-CoV-2 3CLpro with N3, http://doi.org/10.2210/pdb7BQY/pdb), and 2V6N (SARS-CoV 3CLpro with XP-59, http://doi.org/10.2210/pdb2V6N/pdb). Source data for Fig. 1, Fig. 2, Supplementary Fig. 1, and the unprocessed gel for Fig. 1a are available with the paper online.

April 2020

Field-specific reporting

Please select the one below that is the best fit for your research. If you are not sure, read the appropriate sections before making your selection.

- [x] Life sciences
- [ ] Behavioural & social sciences
- [ ] Ecological, evolutionary & environmental sciences

For a reference copy of the document with all sections, see nature.com/documents/nr-reporting-summary-flat.pdf

Life sciences study design

All studies must disclose on these points even when the disclosure is negative.

Sample size	We utilized standard sample sizes used by others for similar assays, such as in Jin et al 2020, Nature and Ma et al 2020, Cell Research. Our data appear in line with the variance in assay results published by other researchers in the field.
Data exclusions	No data were excluded.
Replication	Protease inhibition assays were conducted twice independently with two or three technical replicates each time. Protease kinetics assays were conducted once with two technical replicates. Antiviral assays were conducted twice independently with three technical replicates each time. Independent replicates gave consistent results.
Randomization	Researchers were not blinded to the identity of the compound being tested during the biochemical assay. To control for covariates, control samples and drug treated samples were assayed at the same time. In addition, experiments were performed on two different occasions and the results of testing were averaged to help mitigate the effects of any unknown covariates influencing our assay results. Researchers were blinded for the protease kinetics assay and antiviral assay and compounds were allocated randomly. In these assays, control samples and drug treated samples were assayed at the same time to control for covariates as well.
Blinding	The researcher performing the biochemical assay was not blind to the identify of the compound being tested. To control for bias in interpreting the resulting data, all biochemical results were independently reviewed by another member of the lab along with being repeated twice to ensure their integrity. Testing of compounds in the protease kinetics assay was blinded, with the researcher not informed of the compound that was being tested. Scoring of antiviral assays was blinded, with the scorer not informed of the compound that was being tested.

Reporting for specific materials, systems and methods

We require information from authors about some types of materials, experimental systems and methods used in many studies. Here, indicate whether each material, system or method listed is relevant to your study. If you are not sure if a list item applies to your research, read the appropriate section before selecting a response.

Materials & experimental systems

n/a	Involved in the study	
x		Antibodies
	x	Eukaryotic cell lines
x		Palaeontology and archaeology
x		Animals and other organisms
x		Human research participants
x		Clinical data
x		Dual use research of concern

Methods

n/a	Involved in the study	
x		ChIP-seq
x		Flow cytometry
x		MRI-based neuroimaging

Eukaryotic cell lines

Policy information about cell lines

Cell line source(s)	Vero E6 (ATCC)
Authentication	Cells were received from ATCC with authentication. Morphology was confirmed by microscopy.
Mycoplasma contamination	Cells were confirmed to be mycoplasma contamination negative prior to use.
Commonly misidentified lines (See ICLAC register)	No commonly misidentified lines were used in this study.
